# Metabolic profiling of human colorectal cancer: a top-down approach to translational cancer research

**DOI:** 10.1186/1479-5876-10-S2-A17

**Published:** 2012-10-17

**Authors:** Wei Jia

**Affiliations:** 1 University of North Carolina at Greensboro, North Carolina Research Campus 500 Laureate Way, Suite 4226 Kannapolis, NC 28081, USA

## 

Tumor cells exhibit distinct metabolic phenotypes that are essential for them to sustain higher proliferative rates and resist some cell death signals, altering the flux along key metabolic pathways, such as glycolysis and glutaminolysis. When used as a translational research tool, metabolomics enables the discrimination of distinct metabolic profiles and metabolite markers noninvasively *in vivo* that correlate to pathological stages and different responses to treatment modalities. Cancer metabolomics research aims at evaluating and predicting pathophysiological changes of cancer patients by investigating metabolic signatures in body fluids or tissues, which are influenced by genetics, epigenetics, environmental exposures, diet, and behavior. A particular advantage of metabolomics is that it represents a top-down tactic in that all of the molecules detected are interrogated, providing a global picture of dynamic metabolic changes involving key markers and pathways that were not already associated with carcinogenesis.

We describe here our studies with mass spectrometry based metabolomic profiling of serum, urine and tissue samples from colorectal cancer (CRC) patients. The metabolic profile of CRC involves several significantly altered pathways, including increased glycolysis and an impaired TCA cycle, glutaminolysis, down-regulated urea cycle, dysregulated tryptophan, nucleotides, carnitine, and choline metabolism, and an significantly altered gut microbial-host co-metabolism. Our experimental results highlight the potential for the metabolomic approach to have a multitude of uses in oncology, including the early detection and diagnosis of cancer and as both a predictive and pharmacodynamic marker of therapeutic effect.

